# Psychological treatment of perinatal depression: a meta-analysis

**DOI:** 10.1017/S0033291721004529

**Published:** 2023-04

**Authors:** Pim Cuijpers, Pamela Franco, Marketa Ciharova, Clara Miguel, Lisa Segre, Soledad Quero, Eirini Karyotaki

**Affiliations:** 1 Department of Clinical, Neuro and Developmental Psychology, Amsterdam Public Health Research Institute, Vrije Universiteit Amsterdam, Amsterdam, the Netherlands; 2 Department of Psychology, Pontificia Universidad Católica de Chile, Santiago, Chile; 3 Millennium Institute for Research in Depression and Personality (MIDAP), Santiago, Chile; 4 College of Nursing, University of Iowa, Iowa City, USA; 5 Department of Basic, Clinical Psychology and Psychobiology, Universitat Jaume I, Castellón, Spain; 6 CIBER of Physiopathology of Obesity and Nutrition (CIBEROBN), Madrid, Spain

**Keywords:** Antenatal depression, cognitive behavior therapy, interpersonal psychotherapy, meta-analysis, perinatal depression, postpartum depression, randomized controlled trials

## Abstract

**Background:**

Depression during pregnancy and after the birth of a child is highly prevalent and an important public health problem. Psychological interventions are the first-line treatment and, although a considerable number of randomized trials have been conducted, no recent comprehensive meta-analysis has evaluated treatment effects.

**Methods:**

We used an existing database of randomized controlled trials of psychotherapies for adult depression and included studies aimed at perinatal depression. Random effects models were used in all analyses. We examined the effects of the interventions in the short and long term, and also examined secondary outcomes.

**Results:**

Forty-three studies with 49 comparisons and 6270 participants between an intervention and control group were included. The overall effect size was *g* = 0.67 [95% confidence interval (CI) 0.45~0.89; numbers needed-to-be-treated = 4.39] with high heterogeneity (*I^2^* = 80%; 95% CI 75~85). This effect size remained largely unchanged and significant in a series of sensitivity analyses, although some publication bias was found. The effects remained significant at 6–12 months follow-up. Significant effects were also found for social support, anxiety, functional limitations, parental stress and marital stress, although the number of studies for each outcome was low. All results should be considered with caution because of the high levels of heterogeneity in most analyses.

**Conclusions:**

Psychological interventions are probably effective in the treatment of perinatal depression, with effects that last at least up to 6–12 months and probably also have effects on social support, anxiety, functional impairment, parental stress, and marital stress.

## Introduction

Depression during pregnancy and after the birth of a child is highly prevalent and results in a considerable reduction in quality of life, social functioning (Drury, Scaramella, & Zeanah, 2016), as well as parental and maternal functioning (Bernard, Nissim, Vaccaro, Harris, & Lindhiem, 2018; O'Hara & McCabe, 2013). The negative consequences of perinatal depression extend to infants and children, including diminished cognitive, emotional, behavioral, and physical outcomes (Liu et al., 2017; O'Hara & McCabe, 2013; Stein et al., 2014). Although it is not clear whether the prevalence of depression is actually more common in the postpartum period than in other periods of life (O'Hara & McCabe, 2013), one meta-analysis estimated that about one in seven women in high-income countries and one in 10 women in low-income countries are affected by perinatal depression (Woody, Ferrari, Siskind, Whiteford, & Harris, 2017). The effective treatment of perinatal depression is, therefore critical to public health.

Psychotherapy is recommended as the first-line approach for perinatal women with a new depression episode (O'Connor, Rossom, Henninger, Groom, & Burda, 2016). Importantly, women generally prefer psychotherapy due to concerns about the effects of medication on their infants (Dennis & Chung-Lee, 2006). More than 40 randomized controlled trials have tested the effects of psychological treatments of perinatal depression and several meta-analyses have evaluated overall effects (Bledsoe & Grote, 2016; Dhillon, Sparkes, & Duarte, 2017). Nevertheless, no recent meta-analysis has integrated the results of all published trials. Instead, meta-analytic evaluations have included a subset of trials across a wide range of dimensions including the type of therapy (Cluxton-Keller & Bruce, 2018; Huang, Zhao, Qiang, & Fan, 2018; Lever Taylor, Cavanagh, & Strauss, 2016; Roman, Constantin, & Bostan, 2020; Shortis, Warrington, & Whittaker, 2020; Sockol, 2015, 2018), delivery modalities (Lau, Htun, Wong, Tam, & Klainin-Yobas, 2017; Loughnan, Joubert, Grierson, Andrews, & Newby, 2019; Nair, Armfield, Chatfield, & Edirippulige, 2018; Roman et al., 2020; Stevenson et al., 2010; Zhou et al., 2020), settings (Rahman et al., 2013; Stephens, Ford, Paudyal, & Smith, 2016), and timing i.e. antenatal or postpartum period (Huang et al., 2020; Li et al., 2020a; Roman et al., 2020; van Ravesteyn, Lambregtse-van den Berg, Hoogendijk, & Kamperman, 2017). Additionally, published meta-analyses which focused on broader mental health problems in the perinatal period, such as anxiety and distress in general, resulted in unclear and incomplete effect estimates of depression treatments (Han, Guo, Ren, Duan, & Xu, 2020; Li et al., 2020b; Song, Kim, & Ahn, 2015). Some meta-analyses included non-randomized trials with the associated increase of uncertainty of effect estimates (Sockol, 2015). Finally, despite the link between maternal depression and diminished development in children, no prior meta-analyses of perinatal depression treatments have assessed whether treatment results in improved child outcomes.

With the substantial number of published clinical trials evaluating psychotherapy treatments for perinatal depression, the field has reached a critical point of having adequate statistical power to examine, not only the effect size of these treatments, but also the ability to examine secondary outcomes and potential sources of heterogeneity in subgroup analyses. A larger meta-analysis also allows to examine the relative effectiveness of different treatments, modalities, settings, and timing of treatment.

More studies also provide a more robust estimate of long-term effects, which are often understudied in psychotherapy research. In one earlier larger meta-analysis of all psychotherapies for adult depression, a relatively complete set of studies on psychotherapies in perinatal depression (*N* = 36) were included. However, this analysis only resulted in an overall pooled effect size (Cuijpers, Karyotaki, Reijnders, & Huibers, 2018a). No secondary outcomes, subgroup analyses or long-term effects of these studies were conducted.

The limitations of prior meta-analyses thus prompted a new comprehensive meta-analysis of randomized trials comparing psychological treatments of perinatal depression with control groups. Here we focus not only on the overall pooled effect size of treatments for depressed women, but also on secondary outcomes in mother and child, incorporating subgroup analyses to explore the potential impact of moderators, and long-term effects.

## Methods

### Identification and selection of studies

The protocol for this meta-analysis was registered at the Open Science Framework (Cuijpers, 2020b; Cuijpers & Karyotaki, 2020; https://osf.io/cmk4y). We used an existing database of studies on the psychological treatment of depression. This database has been described in detail elsewhere (Cuijpers & Karyotaki, 2020), and has been used in a series of earlier published meta-analyses (Cuijpers, 2017). The database is continuously updated and was developed through a comprehensive literature search (from 1966 to January 1, 2020). For this database, we searched four major bibliographical databases (PubMed, PsycINFO, Embase and the Cochrane Library) by combining terms (both index terms and text words) indicative of depression and psychotherapies, with filters for randomized controlled trials. The full search string for one database (PubMed) is given in online Supplementary Appendix A. We also searched a number of bibliographical databases to identify trials in non-Western countries (Cuijpers, Karyotaki, Reijnders, Purgato, & Barbui, 2018b), because the number of trials on psychological treatments in these countries is growing rapidly. Furthermore, we checked the references of earlier meta-analyses on psychological treatments of depression. For the current meta-analysis, we also checked the references of previous meta-analyses on the psychological treatment of perinatal depression. All records were screened by two independent researchers and all papers that could possibly meet inclusion criteria according to one of the researchers were retrieved as full-text. The decision to include or exclude a study in the database was also done by the two independent researchers, and disagreements were solved through discussion.

For the current meta-analysis, we included studies that were: (a) randomized trials (b) in which a psychological treatment (c) for perinatal depression (d) was compared with a control group (waiting list, care-as-usual, placebo, other inactive treatment). Perinatal depression was defined as depression during pregnancy (antenatal depression) and up to two years after giving birth (postpartum depression). Depression could be established with a diagnostic interview or with a score above a cut-off on a self-report measure. No language restrictions were applied.

### Quality assessment and data extraction

As in our previous meta-analyses using our database of randomized trials, we assessed the validity of included studies using four criteria of the ‘Risk of bias’ assessment tool, developed by the Cochrane Collaboration (Higgins et al., 2011). This tool assesses possible sources of bias in randomized trials, including the adequate generation of allocation sequence; the concealment of allocation to conditions; the prevention of knowledge of the allocated intervention (masking of assessors); and dealing with incomplete outcome data (this was assessed as positive when intention-to-treat analyses were conducted, meaning that all randomized patients were included in the analyses). Assessment of the validity of the included studies was conducted by two independent researchers, and disagreements were solved through discussion.

We also coded participant characteristics (depressive disorder or scoring high on a self-rating scale; antenatal, postnatal, or mixed; the age of participants); we also rated whether studies were explicitly aimed at high-risk women or not (high risk was defined as low-income and/or ethnic minority); type of therapy (according to the framework developed previously; Cuijpers, Karyotaki, de Wit, & Ebert, 2020a; Cuijpers, van Straten, Andersson, & van Oppen, 2008) and other characteristics of the psychotherapies (treatment format; the number of sessions); and general characteristics of the studies (type of control group; the country where the study was conducted). The treatment format was coded as an individual, group or guided self-help (including internet-based guided self-help). We did not include unguided self-help, because this has been found to be less effective in the treatment of depression (Cuijpers, Noma, Karyotaki, Cipriani, & Furukawa, 2019).

### Outcome measures

For each comparison between psychotherapy and a control condition, the effect size indicating the difference between the two groups at post-test was calculated (Hedges' g) (Hedges & Olkin, 1985). Effect sizes of 0.8 can be assumed to be large, while effect sizes of 0.5 are moderate, and effect sizes of 0.2 are small (Cohen, 1988). Effect sizes were calculated by subtracting (at post-test) the average score of the psychotherapy group from the average score of the control group and dividing the result by the pooled standard deviation. Because some studies had relatively small sample sizes we corrected the effect size for small sample bias (Hedges & Olkin, 1985). If means and standard deviations were not reported, we used the procedures of the Comprehensive Meta-Analysis software (see below) to calculate the effect size using dichotomous outcomes; and if these were not available either, we used other statistics (such as *t* value or *p* value) to calculate the effect size.

In order to calculate effect sizes, we used all measures examining depressive symptoms (such as the Beck Depression Inventory/BDI (Beck, Ward, Mendelson, Mock, & Erbaugh, 1961); the BDI-II (Beck, Steer, & Brown, 1996); or the Hamilton Rating Scale for Depression/HAMD-17 (Hamilton, 1960). When one study reported more than one depression outcome measure, we pooled the effect sizes within the studies before pooling across studies. We also conducted sensitivity analyses in which we used only one depression outcome measure from each study, based on an algorithm that we used in a previous meta-analysis on psychotherapies for depression, giving priority to the HAM-D-17, the BDI, the BDI-II, another clinician-rated instrument and another self-report instrument (Cuijpers et al., 2020b). Effect sizes were calculated using Comprehensive Meta-Analysis (version 3.3070; CMA).

We also extracted secondary outcomes, like the quality of life, social support, functional limitations and child outcomes. We checked in the included studies which secondary outcomes were included and reported, and if 5 or more studies reported on the same outcome, this outcome was included in the meta-analysis. For definitions and operationalizations of secondary outcomes, we used previous meta-analyses of our database on quality of life (Kolovos, Kleiboer, & Cuijpers, 2016), functional limitations (Renner, Cuijpers, & Huibers, 2014), social support (Park, Cuijpers, van Straten, & Reynolds, 2014), and child outcomes (Cuijpers, Weitz, Karyotaki, Garber, & Andersson, 2015).

### Meta-analyses

We pooled the effect sizes using the ‘meta’ and ‘metafor’ packages in R, and ran all analyses in R studio, version 1.1.463, for Mac (the R Foundation). Because we expected considerable heterogeneity among the studies, we employed a random-effects pooling model in all analyses. We pooled the effect sizes using the inverse variance method, with the Hartung-Knapp adjustment for the random-effects model. We calculated the *I^2^* statistic and its 95% confidence interval (CI) to estimate heterogeneity (Higgins, Thompson, Deeks, & Altman, 2003). A value of 0% indicates no observed heterogeneity, and larger values indicate increasing heterogeneity, with 25% as low, 50% as moderate, and 75% as high heterogeneity. In addition, we calculated the prediction interval. Because the 95% CI of the effect size does not indicate how the true effects found in studies are distributed, we added the prediction interval which indicates the range in which the true effect size of 95% of all populations will fall (Borenstein, Hedges, Higgins, & Rothstein, 2009; Borenstein, Higgins, Hedges, & Rothstein, 2017).

Numbers needed-to-be-treated (NNT) for depression outcomes were calculated using the formulae provided by Furukawa (1999). The NNT is the inverse of the risk difference between the treatment and control group (Laupacis, Sackett, & Roberts, 1988), based on a binary outcome. The NNT can be estimated with a continuous outcome, especially if the proportion of a binary outcome is known. In our calculation, we conservatively set the proportion of the outcome at 19% for the control group (based on the pooled response rate of 50% reduction of symptoms across trials in psychotherapy for depression) (Cuijpers et al., 2014). We did not calculate NNTs for other outcomes, because the control group's outcome rates were unknown.

We tested for publication bias by inspecting the funnel plot on primary outcome measures and by Duval and Tweedie's trim and fill procedure (Duval & Tweedie, 2000) as implemented in CMA, which yields an estimate of the effect size after the publication bias has been taken into account. We also conducted Egger's test of the intercept to quantify the bias captured by the funnel plot and to test whether it was significant. Studies were considered to be outliers when the 95% confidence interval of the effect size did not overlap with the pooled effect size.

We conducted subgroup analyses according to the mixed-effects model, in which effect sizes within subgroups are pooled according to the random-effects model and differences between subgroups are tested with a fixed-effects model. We conducted bivariate meta-regression analyses to examine the association between the effect size and continuous outcomes.

We conducted sensitivity analyses in which we included only studies with a low risk of bias, and analyses in which the alternative way of calculating effect sizes was used. We also calculated the relative risk (RR) of dropping out from the study (for any reason) in the intervention groups compared with the control groups.

## Results

### Selection and inclusion of studies

**Fig. 1.**
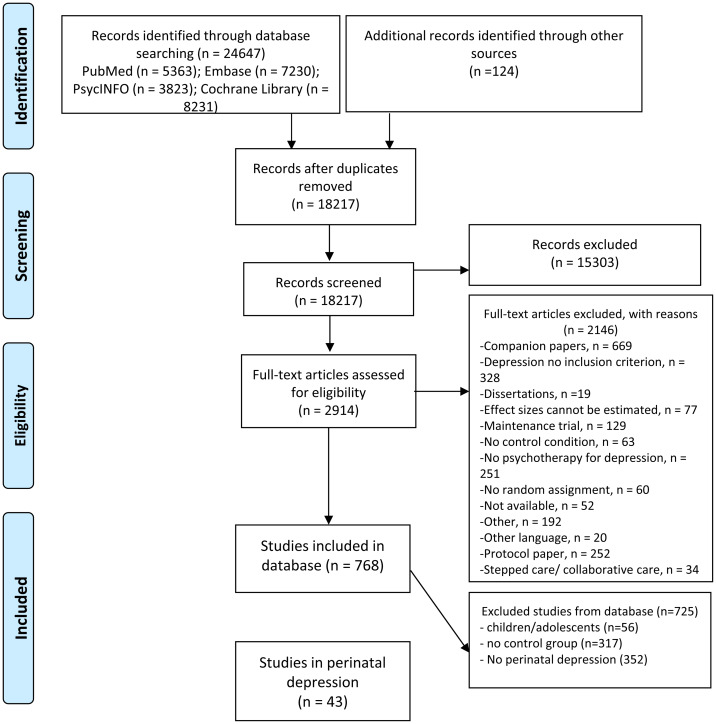
Flowchart on the selection of studies.

After examining a total of 24 771 records (18 217 after removal of duplicates), we retrieved 2914 full-text papers for further consideration. We excluded 2871 of the retrieved papers. The PRISMA flowchart describing the inclusion process, including the reasons for exclusion, is presented in Fig. 1. A total of 43 randomized controlled trials (with 49 comparisons between psychotherapy and a control group) with 6270 participants (3158 in the treatment groups and 3112 in the control groups) met inclusion criteria for this meta-analysis.

### Characteristics of included studies

A summary of key characteristics of the included studies is presented in Table 1. Eighteen of the 43 studies were aimed at pregnant women, 24 at women with postpartum depression and one was aimed at a mixed population. Eight studies were specifically aimed at high-risk women. The mean age of the women in the study populations ranged from 21.9 to 32.2 years (median 29.2; three studies did not report the mean age). In 22 studies women had to meet diagnostic criteria for a depressive disorder to participate, in 21 studies women had to score above the cut-off of a self-rating depression measure.

**Table 1.** Selected characteristics of included studies

			HR	M age		Format	*N* sess					Risk of bias			
Study	Post / ante	Mood			type			*N* psy	Control	*N* ctr	Country	sg	ac	ba	itt
Ammerman et al. (2013)	Mixed	Mood	Y	21.9	cbt	ind	11	47	CAU	46	US	–	+	+	+
Burns et al. (2013)	Ante	Cut-off	N	29.2	cbt	ind	12	16	CAU	13	EU	+	+	sr	+
Chen, Tseng, Chou, and Wang (2000)	Post	Cut-off	N	29.1	sup	grp	4	30	CAU	30	Other	–	–	sr	–
Cho, Kwon, and Lee (2008)	Ante	Mood	N	29.6	cbt	ind	9	12	CAU	10	Other	–	–	sr	–
Clark, Tluczek, and Wenzel (2003)	Post	Mood	N	31.4	ipt	grp	12	15	WL	6	US	–	–	sr	–
					other	grp	12	9	WL	6	US	–	–	sr	–
Clark, Tluczek, and Brown (2008)	Post	Mood	N	30.9	other	grp	12	14	WL	15	US	–	–	sr	–
Cooper, Murray, Wilson, and Romaniuk (2003)	Post	Mood	N	27.7	cbt	ind	10	42	CAU	17	EU	+	+	sr	–
					dyn	ind	10	45	CAU	17	EU	+	+	sr	–
					sup	ind	10	47	CAU	17	EU	+	+	sr	–
Dimidjian et al., (2017)	Ante	Cut-off	N	28.8	bat	other	10	70	CAU	68	US	+	+	sr	+
Forsell, Bendix, and Holländare (2017)	Ante	Mood	N	31.0	cbt	gsh	5	21	CAU	18	EU	+	+	sr	+
Fuhr et al. (2019)	Ante	Cut-off	N	25.2	cbt	ind	10	122	CAU	129	East As.	–	+	+	+
Goodman, Prager, Goldstein, and Freeman (2015)	Post	Cut-off	N	30.7	other	ind	8	21	CAU	21	US	–	+	sr	+
Grote et al. (2009)	Ante	Cut-off	N	24.5	ipt	ind	8	25	CAU	28	US	–	–	sr	–
Holden, Sagovsky, and Cox (1989)	Post	Mood	N	26.0	sup	ind	8	26	CAU	24	EU	+	–	+	–
Honey, Bennett, and Morgan (2002)	Post	Cut-off	N	27.9	cbt	grp	8	23	CAU	22	EU	–	–	sr	+
Hou et al. (2014)	Post	Mood	N	28.0	cbt	other	19	104	CAU	109	Other	–	–	sr	–
Husain et al. (2017)	Post	Mood	Y	27.7	cbt	grp	6	112	CAU	104	East As.	–	+	+	+
Jesse et al. (2015)	Ante	Cut-off	Y	25.1	cbt	grp	6	39	CAU	72	US	+	+	sr	–
Jiang et al. (2014)	Post	Cut-off	N	27.3	other	other	18	257	CAU	514	Other	+	–	sr	–
Lenze and Potts (2017)	Ante	Mood	Y	26.6	ipt	ind	8	21	CAU	21	US	+	+	sr	+
Leung et al. (2013)	Ante	Cut-off	N	NR	cbt	grp	6	47	CAU	50	Other	–	–	sr	+
Leung et al. (2016)	Post	Cut-off	N	31.0	cbt	grp	6	82	Other	82	Other	–	–	sr	–
Lund et al. (2020)	Ante	Cut-off	Y	27.0	other	ind	6	148	CAU	187	East As.	+	+	+	+
McKee et al. (2006)	Ante	Cut-off	Y	24.4	cbt	ind	2	21	CAU	20	US	–	–	sr	–
Milgrom, Negri, Gemmill, McNeil, and Martin (2005)	Post	Mood	N	29.7	cbt	grp	9	31	CAU	6	AUS	+	+	sr	+
					sup	grp	9	34	CAU	6	AUS	+	+	sr	+
					sup	ind	9	38	CAU	6	AUS	+	+	sr	+
Milgrom et al. (2011)	Post	Cut-off	N	31.5	cbt ^a^	ind	5	22	CAU	12	AUS	+	+	sr	+
					cbt ^b^	ind	4	23	CAU	12	AUS	+	+	sr	+
Milgrom et al. (2015)	Post	Mood	N	31.8	cbt	ind	6	23	CAU	21	AUS	+	+	sr	+
Milgrom et al. (2016)	Post	Mood	N	31.6	cbt	gsh	6	21	CAU	22	AUS	+	+	sr	+
Morrell et al. (2009)	Post	Cut-off	N	30.9	sup	ind	8	271	CAU	147	EU	+	+	sr	+
Mulcahy et al. (2010)	Post	Mood	N	32.2	ipt	other	11	23	CAU	27	AUS	+	–	–	–
Ngai, Wong, Leung, Chau, and Chung (2015)	Post	Cut-off	N	30.8	cbt	other	5	197	CAU	200	Other	+	+	sr	+
O'Hara, Stuart, Gorman, and Wenzel (2000)	Post	Mood	N	29.6	ipt	ind	12	48	WL	51	US	+	–	–	+
O'Mahen, Himle, Fedock, Henshaw, and Flynn (2013)	Ante	Mood	Y	27.1	cbt	ind	8	30	CAU	25	EU	+	+	sr	+
Prendergast and Austin (2001)	Post	Mood	N	32.2	cbt	ind	6	17	Other	20	AUS	+	–	–	–
Puckering, McIntosh, Hickey, and Longford (2010)	Post	Cut-off	N	NR	other	grp	14	10	WL	4	EU	+	–	sr	–
Pugh, Hadjistavropoulos, and Dirkse (2016)	Post	Cut-off	N	30.8	cbt	gsh	6	21	WL	20	CAN	+	+	sr	–
Rahman, Malik, Sikander, Roberts, and Creed (2008)	Ante	Mood	N	267	cbt	grp	16	412	Other	386	East As.	+	+	+	–
Sikander et al. (2019)	Ante	Cut-off	Y	27.0	cbt	other	11	223	CAU	211	East As.	+	+	+	+
Spinelli and Endicott (2003)	Ante	Mood	N	28.8	ipt	ind	16	21	Other	17	US	–	–	–	+
Spinelli et al. (2012)	Ante	Mood	N	29.5	ipt	other	12	43	Other	37	US	+	–	–	–
Wickberg and Hwang (1996)	Post	Mood	N	28.4	sup	ind	6	15	CAU	16	EU	–	–	+	–
Wiklund, Mohlkert, and Edman (2010)	Post	Cut-off	N	NR	cbt	ind	21	33	CAU	34	EU	–	–	sr	–
Zemestani, Davoodi, Honarmand, Zargar, and Ottaviani (2016)	Ante	Mood	N	29.6	3rd	grp	8	19	CAU	19	East As.	+	+	sr	+
Zhao, Munro-Kramer, Shi, Wang, and Zhao (2019)	Ante	Cut-off	N	30.5	other	grp	4	167	CAU	167	Other	+	–	sr	–

Abbreviations (alphabetical): –, unclear of high risk of bias; +, low risk of bias; 3rd, third-wave therapies; ac, allocation concealment; AUS, Australia; ba, blinded assessment; bat, behavioral activation therapy; CAN, Canada; CAU, care-as-usual; cbt, cognitive behavior therapy; dyn, psychodynamic therapy; East As, East Asia; EU, Europe; grp, group therapy; HR, high-risk; ind, individual therapy; ipt, interpersonal psychotherapy; ITT, intention-to-treat analyses; M age, mean age; N ctr, number of participants in control condition; N psy, number of participants in therapy condition; N sess, number of sessions; N, no; NR, not reported; sg, sequence generation; sr, self-report; sup, supportive therapy; US, United States; WL, waiting list; Y, yes.

a Nurse-led.

b Psychologist-led.

In the 43 studies, 49 psychological interventions were compared with a control group. Twenty-four of these were cognitive behavior therapy, seven were interpersonal psychotherapy, seven were supportive counseling, and 10 were other therapies. Twenty-four of the 49 interventions used an individual format, 15 a group format, three internet-based guided self-help, and seven used a mixed or another format. Thirty-six interventions had between 6 and 12 sessions, seven had fewer sessions, and six had more sessions (range 2–21).

A total of 33 studies used a care-as-usual as a control group, five used a waiting list and the other five studies used another control group. Thirteen studies were conducted in North America, 10 in Europe, six in Australia, six in East Asia and eight in other countries.

The risk of bias in many studies was considerable. Twenty-six of the 43 studies reported an adequate sequence generation (61%). Twenty-two reported allocation to conditions by an independent (third) party (51%). Eight studies reported using blinded outcome assessors (19%), and 30 used only self-report outcomes (70%). In 22 studies intent-to-treat analyses were conducted (51%). Fourteen of the 43 studies (33%) met all quality criteria, 15 studies (35%) met two or three of the criteria and the 14 remaining studies met no or only one criterion (33%).

### Overall effects of psychological treatments of perinatal depression

**Fig. 2.**
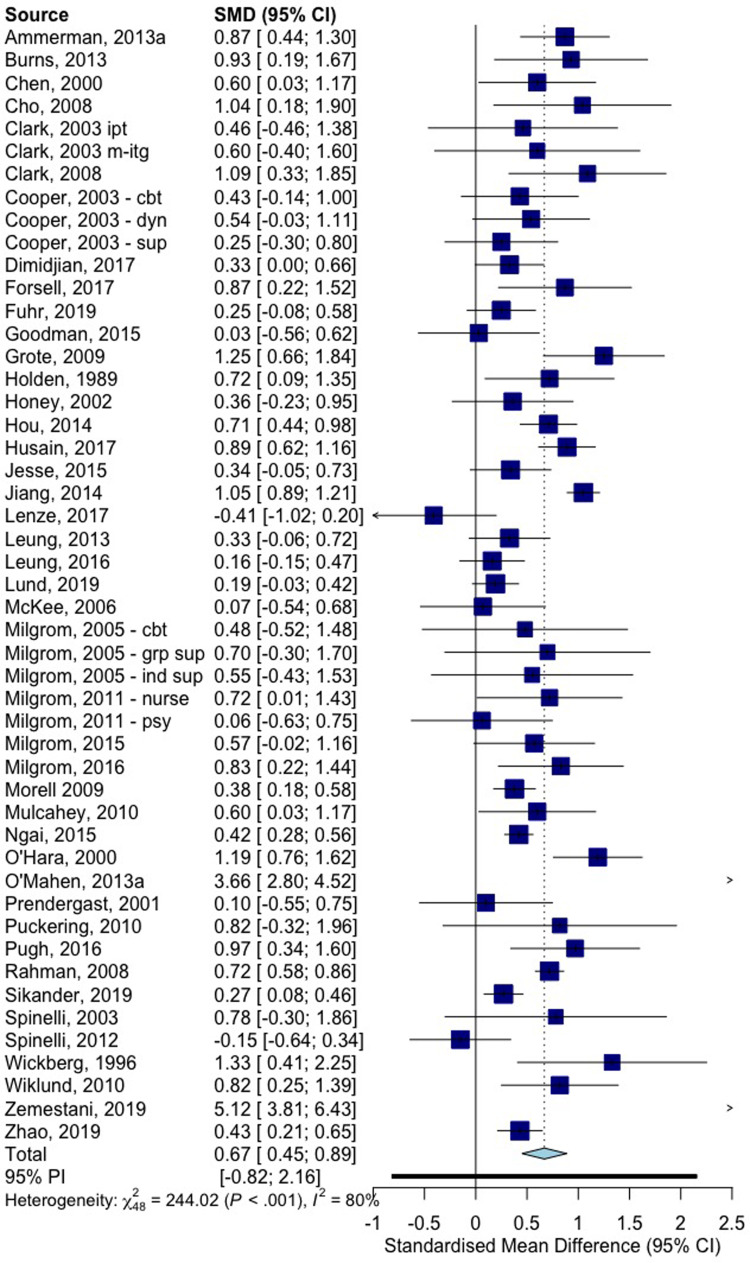
Forest plot of studies comparing psychotherapy for perinatal depression with control conditions: Hedges' g.

The mean effect size of all 49 comparisons of psychotherapy with a control group was *g* = 0.67 (95% CI 0.45~0.89), which corresponds with an NNT of 4.39. Heterogeneity was very high (*I^2^* = 80%; 95% CI 75~85), and the prediction interval ranged from −0.82 to 2.16. The data of these analyses are presented in Table 2 and Fig. 2 gives the forest plot.

**Table 2.** Effects of psychological treatments of perinatal depression: Hedges' g ^a^

		*N_comp_*	*g*	95% CI	*I*^2^	95% CI	Prediction interval	*NNT*	*p*
All comparisons		49	0.67	0.45−0.89	80	75–85	−0.82 to 2.16	4.39	
One ES / study (highest)		43	0.71	0.47–0.96	83	77–87	−0.88 to 2.31	4.11	
One ES / study (lowest)		43	0.69	0.43–0.94	83	78–87	−0.93 to 2.31	4.25	
Outliers excluded ^b^		43	0.56	0.46–0.66	48	25–63	0.06–1.06	5.40	
Only low risk of bias		17	0.87	0.20–1.54	87	81–91	−1.91 to 3.64	3.26	
Adjusted for publication bias		53	0.53	0.24–0.82	85	81–88	−1.56 to 2.62	5.75	
Only EPDS		22	0.45	0.30–0.59	47	13–68	−0.16 to 1.05	6.93	
	*Mean difference*		2.15	1.40–2.90	71	56–81	−1.00 to 5.30		
Only HAMD-D		5	0.71	0.13–1.30	90	80–95	−0.80 to 2.23	4.11	
	*Mean difference*		4.82	0.70–8.94	93	87–96	−5.95 to 15.59		
Only BDI-II		11	1.14	0.08–2.20	91	85–94	−2.51 to 4.80	2.42	
	*Mean difference*		7.04	2.37–11.70	94	91–96	−8.45 to 22.52		
Only BDI		5	0.91	0.49–1.34	0	0–70	0.09–1.74	3.10	
	*Mean difference*		8.32	4.90–11.74	0	0–68	1.61–15.03		
Subgroup analyses									
• Ante/postnatal	− Antenatal	18	0.83	0.19–1.47	89	84–92	−1.92 to 3.58	3.44	0.42
	− Postnatal	30	0.61	0.49–0.73	64	47–76	0.10–1.12	4.89	
	− Mixed	1	0.87	0.44–1.10		^c^	^c^	3.26	
• Diagnosis	− Mood disorder	27	0.86	0.46–1.26	80	72–86	−1.17 to –2.90	3.30	0.05
	− Above cut-off	22	0.46	0.31–0.60	77	65–84	−0.14 to –1.05	6.76	
• Risk status	− High risk	8	0.71	−0.31 to 1.72	91	86–95	−2.38 to –3.79	4.11	0.92
	− No high risk	41	0.66	0.45–0.86	74	64–81	−0.62 to –1.94	4.47	
• Format	− Individual	24	0.65	0.34–0.96	78	68–85	−0.80 to –2.10	4.55	0.02
	− Group	15	0.81	0.20–1.41	80	67–87	−1.55 to –3.16	3.53	
	− Internet-based	3	0.89	0.71–1.07	0	0–0	0.33–1.45	3.18	
	− Mixed/other	7	0.48	0.14–0.83	90	83–95	−0.46 to –1.43	6.44	
• Type	− CBT	25	0.64	0.38–0.90	77	66–84	−0.62 to –1.90	4.63	0.78
	− IPT	7	0.53	−0.08 to 1.13	81	62–91	−1.06 to –2.11	5.75	
	− Supportive	7	0.54	0.27–0.82	0	0–70	−0.09 to –1.18	5.63	
	− Other	10	0.95	−0.05 to 1.95	91	85–94	−2.33 to –4.24	2.95	
• Control group	− Waiting list	6	0.97	0.68–1.25	0	0–55	0.43–1.50	2.88	0.01
	− Care as usual	38	0.70	0.42–0.98	82	77–87	−1.00 to –2.39	4.18	
	− Other	5	0.31	−0.18 to 0.80	82	57–92	−0.86 to –1.48	10.52	
• Country	− North America	14	0.52	0.21–0.82	71	50–83	−0.51 to –1.54	5.88	0.45
	− Europe	12	0.89	0.33–1.45	82	69–89	−1.04 to –2.81	3.18	
	− Australia	9	0.51	0.29–0.72	0	0–42	0.08–0.94	6.01	
	− Other	14	0.79	0.14–1.44	90	85–94	−1.74 to –3.32	3.64	
• Risk of bias	− Lower	27	0.72	0.32–1.12	84	77–88	−1.35 to –2.79	4.04	0.65
	− Higher	22	0.62	0.44–0.81	71	56–81	−0.08 to –1.33	4.80	
Long term outcomes									
FU closest to 12 months		14	0.40	0.17–0.63	76	60–86	−0.35 to –1.15		
Adjusted for publication bias		17	0.54	0.30–0.77	80	69–87	−0.34 to –1.42		
Outlier excluded ^d^		13	0.40	0.15–0.65	78	62–87	−0.40 to –1.20		
One ES / study (highest)		12	0.49	0.26–0.72	73	51–85	−0.21 to –1.19		
One ES / study (lowest)		12	0.47	0.22–0.72	75	57–86	−0.28 to –1.23		
Only low Risk of bias		4	0.46	−0.02 to 0.94	16	0–87	−0.76 to 1.68		
FU between 6 and 12 months		13	0.41	0.17–0.64	75	57–86	−0.34 to 1.16		
FU between 13 and 24 months		4	0.27	−0.77 to 1.32	93	86–97	−2.76 to 3.31		

Abbreviations: CBT, cognitive behavior therapy; CI, confidence interval; ES, effect size; FU, follow-up; IPT, interpersonal psychotherapy; *N*_comp_, number of comparisons; NNT, numbers-needed-to-be-treated.

a According to a random effects model.

b Outliers were: Jiang et al., 2014; Lenze & Potts, 2017; Lund et al., 2020; O'Mahen et al., 2013; Spinelli et al., 2012; Zemestani et al., 2016.

c The 95% CI of I2 and the prediction interval cannot be calculated when the number of studies is less than 3.

d Hou et al., 2014.

There were two studies in which two psychological treatments were compared with the same control group, and two more studies in which three interventions were compared with the same control group. These effect sizes are not independent of each other and may artificially reduce heterogeneity and affect the effect size. Therefore, we conducted two sensitivity analyses, one in which we only included the largest of the two effect sizes from these five studies, and another one in which we included only the smallest effect size. As can be seen in Table 2, these analyses did not point at major differences of the effect sizes or the level of heterogeneity.

After removal of six outliers (the confidence interval of the effect size did not overlap with the pooled effect size; Table 2), the effect size dropped somewhat (*g* = 0.56; 95%: 0.46−0.66), and heterogeneity dropped considerably, but remained moderately high (*I^2^* = 48; 95% CI 25−63). The 17 studies with a low risk of bias resulted in a somewhat higher effect size (*g* = 0.87; 95% CI 0.20~1.54), but heterogeneity was still very high (*I^2^* = 87; 95% CI 81–91). We found some indications for significant publication bias. Egger's test was not significant (*p* = 0.33), but Duval and Tweedie's trim and fill procedure identified four missing studies. After adjustment for these missing studies, the effect size dropped to *g* = 0.53 (95% CI 0.24–0.82). The funnel plot is presented in online Supplementary Appendix B.

The effect sizes found for specific outcome questionnaires ranged from *g* = 0.45 (95% CI 0.30–0.59) for the EPDS to *g* = 1.14 (95% CI 0.08–2.20) for the BDI-II. The mean differences between treatment and control groups, indicating the exact number of points on the scales, were 2.15 for the EPDS, 4.82 for the HAMD, 7.04 for the BDI-II and 8.32 for the BDI.

### Subgroup analyses, long-term outcomes and acceptability

We conducted a series of subgroup analyses (Table 2). We did not find a significant difference between the effect sizes found for period (antenatal, postnatal, mixed), high-risk group *v.* no high-risk group, type of therapy, country where the study was conducted or higher *v.* lower risk of bias. We did find that studies in which participants had to meet diagnostic criteria for a depressive disorder had larger effect sizes than studies in which participants scored above a cut-off on a self-rating scale (*p* < 0.05). We also found a differential effect size for different treatment formats, where a mixed-format had the lowest effects (*p* = 0.02), and we found that type of control group was associated with the effect size, with waiting lists resulting in the largest effect sizes. Heterogeneity remained high in all subgroups with a substantial number of studies.

Fourteen studies reported outcomes at 6 months or longer follow-up. The results are summarized in Table 2. When we took from each study the effect size that was closest to 12 months follow-up, the pooled effect size was *g* = 0.40 (95% CI 0.17–0.63), with high heterogeneity (*I^2^* = 76; 95% CI 60–86). When we limited that to the follow-up measures between 6 and 12 months, the effect size was very comparable (*g* = 0.41). Four studies reported effect sizes at follow-up between 13 and 24 months, but this was not significant. Two studies reported effect sizes longer than 24 months, but we did not pool these. The sensitivity analyses with long-term outcomes were very comparable to the main results (Table 2).

We could calculate study drop-out for psychotherapy and control conditions in 43 comparisons, but we found no indication for a differential drop-out rate in treatment or control groups (RR = 1.11; 95% CI 0.86–1.42; *p* = 0.41).

### Effects of psychotherapies for perinatal depression on other outcomes

The effects of psychotherapies for perinatal depression on other outcomes are reported in Table 3. Forest plots and funnel plots for the secondary outcomes are given in online Supplementary Appendix C. We found significant effects of treatment on social support (*g* = 0.41; 95% CI 0.10–0.72). However, after adjustment for publication bias, these results were no longer significant, and the studies with low-risk bias also did not show positive effects.

**Table 3.** Effects of psychological treatments of perinatal depression on other outcomes: Hedges' g^a^^,^^b^^,^^c^^,^^d^

	*N _comp_*	*g*	95% CI	*I*^2^	95% CI
Social support					
All studies	12	0.41	0.10–0.72	68	42–83
Outliers excluded	11	0.28	0.17–38	20	0–60
Only low risk of bias	6	0.62	−0.14– to 1.38	82	61–92
Adjusted for publication bias	14	0.28	−0.11–0.68	78	63–87
Anxiety					
All studies	11	0.71	0.08–1.34	82	69–90
Outliers excluded	10	0.47	0.16–0.79	58	15–79
Only low risk of bias	8	0.85	0.02–1.68	83	67–91
Adjusted for publication bias	14	0.35	−0.38 to –1.07	88	82–92
Functional impairment					
All studies	7	0.46	0.09–0.83	88	78–94
Adjusted for publication bias	8	0.38	0.01–0.75	88	79–93
Only low risk of bias	3	0.12	0.04–0.20	0	0–0
Parental stress					
All studies	7	0.68	0.49–0.87	0	0–69
Only low risk of bias	2	0.51	0.27–0.75	x	X
Adjusted for publication bias	10	0.53	0.31–0.75	50	0–76
Marital stress					
All studies	5	0.57	−0.10 to –1.24	63	2–86
Adjusted for publication bias	7	0.30	−0.43 to –1.02	73	43–88
Weight					
All studies	5	0.11	−0.10 to –0.32	55	0–83
Only low risk of bias	2	0.06	−1.58 to –1.69	x	X
Adjusted for publication bias	7	0.01	−0.21 to –0.23	73	41–87
Height					
All studies	5	0.10	−0.01 to –0.21	0	0–72
Only low risk of bias	2	0.08	−0.04 to –0.19	x	x

a According to a random-effects model.

b This sample of studies did not include studies with a low risk of bias.

c The two outliers in the main analyses were excluded (Hsu et al., 2009; Tsai et al., 2008).

d The 95% CI of *I*^2^ and the prediction interval cannot be calculated when the number of studies is less than 3

Underlined values are significant (p<0.05)

We also found significant effects on anxiety (*g* = 0.71; 95% CI 0.08–1.34), which remained significant when outliers were excluded and when only studies with low risk of bias were included. However, after adjustment for publication bias only a moderate, non-significant effect size remained.

We also found significant effects for functional impairment, parental stress and marital stress, also after adjustment for publication bias. Unfortunately, there were not enough studies with a low risk of bias to validate the robustness of these results in high-quality research.

No significant effects were found on the weight and height of the children.

## Discussion

We conducted a comprehensive meta-analysis of all randomized trials examining the effects of psychological interventions for perinatal depression compared to control conditions and also included important secondary outcomes, outcomes at follow-up, as well as child outcomes. We found that the effects of the interventions on depression were moderate to large, and they remained significant after a series of sensitivity analyses. There was some publication bias, but after adjustment for that, the effects were somewhat smaller, but still significant. These effects remained significant at one-year follow-up, although the number of studies with a low risk of bias was small at follow-up, so these results should be considered with caution.

One major problem was the very high level of heterogeneity in most analyses. This means that the effect sizes of the included studies do not point in the same direction. We found several important outliers with effect sizes that are so large that they lack credibility. But even after excluding these extreme outliers, the level of heterogeneity remained considerable. Subgroup analyses pointed at some significant differences between samples of studies. It is known from other meta-analytic research that the type of control group is associated with differential effect sizes (Cuijpers, Quero, Papola, Cristea, & Karyotaki, 2021), and that was confirmed in this study. But we also found that treatment format may be related to the effect size, which is not confirmed in other meta-analytic research (Cuijpers et al., 2019). The same is true for the differential effects of studies in women with a diagnosed mood disorder compared to a score above the cut-off of a self-report depression measure. This has also not been confirmed in other meta-analytic research (Cuijpers, Karyotaki, Reijnders, & Huibers, 2018a). However, the number of studies in subgroups was relatively small, and may very well be chance findings, because moderator analyses need large sample sizes, especially when heterogeneity is high (Hempel et al., 2013). Because the *p* values are not that convincing, and because of potential confounding, these results should be considered with caution.

Apart from the effects on depression, we found indications that the interventions also had positive effects on social support, anxiety, functional limitations, parental stress and marital stress. This is encouraging and is in line with a growing body of research showing that psychological interventions affect not only depression, but also have positive effects on a range of secondary outcomes (Cuijpers, 2020a, 2020b). These results should, however, also be considered with caution because of the risk for publication bias and because each of the outcomes was only examined in relatively small samples of studies, most of which did not meet the criteria for high quality.

We could include a considerable number of studies in high-risk women, with low incomes or from minority groups. It was encouraging to find that the effects of the interventions did not differ for studies in these groups compared to the effects found in other groups.

The results of this meta-analysis are in line with the results of previous meta-analyses, which mostly focused on subsamples of studies, and resulted in very uncertain outcomes. The current meta-analysis indicated more robust and precise outcomes, although the results remained problematic because of the high level of heterogeneity, publication bias, and the low quality of many included trials.

This study has important implications. First of all, it confirms that psychological treatments should be first-line treatments of perinatal depression. These treatments are effective, also in the longer term. Furthermore, they do not only affect depression, but also important secondary outcomes, such as anxiety, social support, functional impairment, parental stress and marital stress. However, the high heterogeneity also indicates that the effect of treatments vary considerably across studies and it is not clear what the causes are of these differences. This means that more and better research is needed to examine who benefits from which treatment under which conditions. One of the causes of the heterogeneity is certainly the fact that many different outcome measures are used and standardization of outcome measures across studies is certainly recommended.

The strengths of the current study include the incorporation of trials spanning the entire perinatal period, the evaluation of other outcomes than depression alone, child outcomes, follow-up outcomes, and state-of-the-art meta-analytic approaches. However, there are also several important limitations that have to be mentioned. We already mentioned the high level of heterogeneity, publication bias, and the low quality of many included trials. In addition to that, it is important to mention that the secondary outcomes were not very consistent across trials, and it would be good if researchers would find some consensus on what secondary outcomes are relevant and should be included in trials. We also included only a limited number of studies reporting longer-term outcomes. The effects in the longer term are highly relevant from a clinical and public health perspective.

Despite these limitations, we can conclude that psychological interventions are effective in the treatment of perinatal depression, with effects that last at least up to 6–12 months and probably also has effects on social support, anxiety, functional impairment, parental stress and marital stress.

## Supplementary material

For supplementary material accompanying this paper visit https://doi.org/10.1017/S0033291721004529. click here to view supplementary material
